# Predicted Studies of Branched and Cross-Linked Polyurethanes Based on Polyhydroxybutyrate with Polycaprolactone Triol in Soft Segments

**DOI:** 10.3390/polym12051068

**Published:** 2020-05-07

**Authors:** Joanna Brzeska, Agnieszka Tercjak, Wanda Sikorska, Marek Kowalczuk, Maria Rutkowska

**Affiliations:** 1 Department of Commodity Industrial Science and Chemistry, Gdynia Maritime University, 83 Morska Street, 81-225 Gdynia, Poland; m.rutkowska@wpit.umg.edu.pl; 2 Department of Chemical and Environmental Engineering, Group ‘Materials+Technologies’ (GMT), University of the Basque Country (UPV/EHU), Plaza Europa 1, 20018 Donostia-San Sebastián, Spain; agnieszka.tercjaks@ehu.eus; 3 Centre of Polymer and Carbon Materials, Polish Academy of Sciences, 34 M. Curie-Sklodowska Street, 41-819 Zabrze, Poland; marek.kowalczuk@cmpw-pan.edu.pl

**Keywords:** polyurethane structure, branched and cross-linked polyurethanes, synthetic polyhydroxybutyrate, polycaprolactone triol, chemical structure, water affinity, thermal and mechanical properties, surface topography

## Abstract

The number of cross-links in the non-linear polyurethane structure is the basic factor affecting its properties. Selected properties of aliphatic polyurethanes with soft segments made of different amounts of polycaprolactonetriol, polycaprolactonediol and synthetic, telechelic poly([R,S]-3-hydroxybutyrate) were determined. On the basis of changes in polyurethane properties, the correlation between these properties and the construction of soft segments was found. The structure of polyurethanes, their morphology, hydrophilicity, thermal and mechanical properties were examined. These properties were changed linearly up to 15% content of polycaprolactonetriol in soft segments. A further increase in the amount of triol causes that these properties are mainly determined by the high number of cross-links.

## 1. Introduction

Polyurethanes are versatile materials, and due to their easy modification, can be used in many industries. Considering the theoretical structure of branched and cross-linked polyurethanes, their properties are mainly affected: (i) By the characteristics of substrates used for their building (such as hydrophilicity, crystallinity, degradability, etc.); (ii) by the molecular weight and the degree of polymerization; (iii) by the degree of branching/cross-linking and length of chains between branching/cross-linking nodes; (iv) and by the number of short and long side chains. These parameters affect the ability to form hydrogen bonds, and this influences almost all properties of the material: Its crystallinity, mechanical strength, wettability, sorption properties, degradability and many others. However, the above-mentioned parameters work synergistically and properties of the obtained materials are the effect of the influence of all of them.

The fundamental way to obtain polyurethane with the desired properties is to select the appropriate substrates for its synthesis. A method to obtain environmentally friendly or compatible with a living organism polyurethane can be to use a natural compound for its synthesis, e.g., polyhydroxybutyrate (PHB). PHB is the most popular compound among all polyhydroxyalkanoates due its biocompatibility, biodegradability and natural origin [1,2,3]. However, despite the undoubted benefits of using PHB, its high crystallinity, brittleness, high production costs, poor mechanical properties and thermal instability cause that its use is limited. A few methods are employed to improve PHB characteristics, such as: Drawing and thermal treatment, blending with other biopolymers and synthetic polymers, forming composites with natural fibers and inorganic fillers and others [4]. However, it is beneficial to replace natural PHB with its synthetic analogue, which is also biocompatible and biodegradable, but possess R,S configuration on β-carbon. Synthetic PHB is synthetized via polymerization of β-butyrolactone (4-methyl-2-oxetanone), using coordination, cationic or anionic ring-opening polymerization, and depending on the kind of monomer and polymerization mechanism can be obtained in R, S or R,S configuration [5]. If PHB is obtained as poly([R,S]-3-hydroxybutyrate) (R,S-PHB) its character is almost completely amorphous. Through the appropriate method of modification it can be formed into telechelic polyol with hydroxyl groups at the ends of its chains [6], and can be used to build soft segments of polyurethanes. The presence of amorphous R,S-PHB with a very short side chain (CH_3_) in the polyurethane structure should influence its properties, such as a change of crystallinity, which facilitates water migration into the polyurethane network, and others. 

Due to the wide spectrum of potential applications of polymer biomaterials, e.g., in medicine, for the production of compostable polymer packaging (such as cosmetics or food), as well as in agrochemical preparations, forensic engineering on advanced polymeric materials (FEAPM) can provide basic knowledge and valuable information to prevent future problems. FEAPM deals with the knowledge and evaluation of relationships between the structure, properties and behavior of polymer materials before, during and after practical applications. This approach also allows predicting the properties of new polyurethanes depending on their structure and composition of the soft segments. In paper the correlation between the quantity of polycaprolactone triol in soft segments and properties of branched and cross-linked polyurethanes based on poly([R,S]-3-hydroxybutyrate) diol were presented. The paper is a continuation of previous research on the impact of linear and branched polyurethane structures on their properties [7]. Here, three series of polyurethanes, different with regard to their amount of polycaprolactone triol and poly([R,S]-3-hydroxybutyrate) diol were synthetized and characterized.

## 2. Experimental

### 2.1. Materials

Poly([R,S]-3-hydroxybutyrate) diol was synthetized via polymerization of β-butyrolactone (Aldrich, St. Luis, MO, USA), using anionic ring-opening polymerization, initiated by 3-hydroxybutyric acid sodium salt (Aldrich, St. Luis, MO, USA)/18-crown-6 complex (Fluka, Germany), at room temperature and terminated with 2-iodoethanol or 2-bromoethanol (Aldrich, St. Luis, MO, USA) [6]. Oligomerols of R,S-PHB (M_n_ 2100), PCL_triol_ (*M*_n_ 900, Aldrich, St. Luis, MO, USA) and PCL_diol_ (*M*_n_ 1900, Aldrich, St. Luis, MO, USA) were dried by heating at 70 °C under reduced pressure (1.4 hPa). 4,4’-methylene dicyclohexyl diisocyanate (H_12_MDI) (Aldrich, St. Luis, MO, USA) was vacuum distilled; 1,4-butanediol (1,4-BD) (Aldrich, Steinheim, Germany) was distilled azeotropically with benzene; *N,N*-dimethylformamide (DMF) (Chempur, Gliwice, Poland) was dehydrated over diphosphorous pentoxide (P_2_O_5_) and distilled under low pressure. Catalyst tin(II) octanoate (OSn) (Alfa Aestar, Karlsruhe, Germany) was used as received.

### 2.2. Methods

#### 2.2.1. Chemical Structure

Attenuated Total Reflectance Fourier Transform Infrared Spectroscopy (ATR FTIR) was used to determine the characteristic groups of polyurethanes. FTIR spectra were recorded with an attenuated total reflection (ATR, Smart Orbit Accessory, Thermo Scientific, Madison, WI, USA) mode on a NICOLET 380 FTIR spectrometer (Thermo Scientific, Madison, WI, USA) with a diamond cell. Resolution of 4 cm^−1^ and the scanning range from 400 to 4000 cm^−1^ were applied, and 32 scans were taken for each measurement.

#### 2.2.2. Surface Topography

Atomic Force Microscopy (AFM) images were obtained with a Nanoscope V scanning probe microscope (Multimode 8, Digital Instruments, Bruker, Billerica, MA, USA) under ambient conditions. Tapping Mode (TM) was employed in air using an integrated tip/cantilever (125 m in length with ca. 300 kHz resonant frequency). Typical scan rates during recording were 0.7 to 1 line/s using a scan head with the maximum range of 15 μm × 15 μm. Some samples were cut using an ultramicrotome Leica Ultracut R with a diamond blade to obtain a sample cross section. 

Optical Microscopy (OM) micrographs were taken using a Nikon Eclipse E600W microscope (Mettler FP 82 HT, Melvile, NY, USA). These micrographs were collected with the software analySIS docu FIVE.

#### 2.2.3. Thermal Properties

Differential Scanning Calorimetry (DSC) measurements were performed using a Mettler Toledo DSC3+ (Mettler Toledo, Columbus, OH, USA). All the investigated samples were first heated from −80 to 190 °C at 10 °C·min^−1^, then cooled from 190−80 °C at 10 °C·min^−1^ and finally, they were melted by heating up to 190 °C at 10 °C·min^−1^. All the experiments were conducted under a nitrogen flow of 10 mL/min using 5 to 10 mg samples in aluminum pans. 

Thermogravimetric Analysis (TGA) was performed with TGA/SDTA-851e equipment (Mettler Toledo, Columbus, OH, USA) under air atmosphere at the heating rate of 10 °C·min^−1^ from room temperature to 800 °C.

#### 2.2.4. Water Contact Angle Measurements

Static water contact angle measurements were carried out using the Data Physics OCA 20 contact angle system (SEO Phoenix 300, Suwon, Korea) at ambient temperature. A 5 mL distilled water drop was used for each measurement and a photo of the drop was taken after 0, 1 and 3 min from immersing the drop on the sample surface. At least five measurements were made for each different system.

#### 2.2.5. Density

The density of polymer samples was determined according to the ISO 1183 standard and using analytical balance equipped with a density determination kit (Radwag, AS 160.X2, Radom, Poland).

Oil sorption and water sorption were measured according to ASTM D2842 (equivalent to ISO 2896) [8]. Samples were dried for 3 h at 40 °C and then weighed. The samples were immersed in oil or distilled water to the depth of 1 cm. PURs were immersed in sunflower oil at 37 °C for 24 h and next they were weighed after wiping off the oil with filter paper.

For water sorption estimation, the investigated polymers were immersed in deionized water for 21 days at 37 °C. Periodically, these specimens were removed from the solvent and weighed after removing an excess of the solvent from the sample using filter paper.

Sorption was calculated (using the gravimetric method) from the weight after incubation (*w*_i_) and the initial weight (*w*_0_) by:

Sorption% = (*w*_i_ − *w*_0_)/*w*_0_ × 100%.

The results were the average of three measurements.

#### 2.2.6. Mechanical Properties

Test Machine MultiTest-1xt (Mecmesin, Slinfold, West Sussex, UK) was used to estimate tensile strength (σ_max_) of the obtained samples. The number of replicates for this test was five. Hardness of the samples was determined by the Shore A method, using HAD 100-1 apparatus (Sauter).

## 3. Results and Discussion

**Figure 1:**
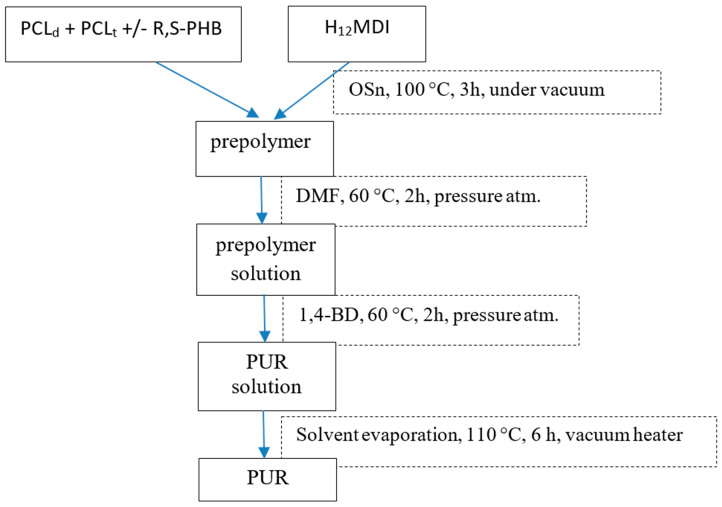
Scheme of PURs synthesis.

Polyurethanes (PURs) were obtained through the prepolymer method detailed in our previous work [7,9]. The diagram of PURs synthesis is presented in Figure 1.

Three series of polyurethanes, with different amount of PCL_t_, were obtained. Polyurethanes in each series differed in the construction of soft segments. Hard segments were synthetized with H_12_MDI and 1,4-BD in each case. The molar ratio of NCO:OH in prepolymer was always 2:1, whereas the total ratio in PUR was 1:1. 

The chemical composition of synthetized PURs is presented in Table 1. PUR series are named according to percentage of oligomerol in soft segments (wt %) of the used R,S-PHB/PCL_t_ system.

Percentage content of side CH_3_ (from R,S-PHB) and side C_2_H_5_ (from PCL_t_) was estimated according to [10] and is given in Table 1. As C_2_H_5_ is attached to the carbon at the branching point of PCL_t_, an increase in its amount in the PUR structure indicates an increase in the degree of branching. Cross-linking and branching density of the polymer network is also expressed through the calculated molecular weight of chains between the branching/cross-linking point (*M*_c_) [11], and is calculated according to [10].

Considering starting substrates used for the synthesis of polyurethanes, it can be assumed that their properties will be influenced by the type and the amount of polyols used, and this will also affect the structure of the polymer: Chain length between branching/cross-linking nodes, and the number of short methyl and ethyl chains, which are a steric hindrance [10].

An increase in the amount of PCL_t_ in soft segments decreases the chain length between branch nodes, i.e., an increase in M_c_. At the same time the number of ethyl side groups increases. Whereas, when the amount of R,S-PHB increases, the number of side methyl groups increases simultaneously.

The synthetized polyurethanes were almost transparent, and those based on R,S-PHB obtained from iodoethanole were slightly yellow (Table 2). Their density was about 1.1 g/cm^−3^, independently of the amount of the added triol. Polyurethanes, obtained with 5 wt % of PCL_t_, were soluble in DMF, which indicated that they were branched; whereas PURs based on the higher amount of PCL_t_ swelled in DMF, due to the formation of the cross-linked structure. However, it did not affect the oil sorption of PURs, which stays at the same level for all samples studied.

The chemical structure of the synthesized PURs was confirmed via FTIR spectroscopy (Figure S1, Supplementary Materials). The characteristic absorption bands which appeared in the FTIR spectra and proved the urethane linkage formation were: A broad absorption band centered at about 3350 cm^−1^, which corresponded to –NH groups (–NH stretching vibrations), the peak corresponding to C=O stretching vibrations observed around 1720 cm^−1^, and peaks of an amide II band and an amide III band around 1520 and 1240 cm^−1^, respectively (Table S1, Supplementary Materials). On FTIR spectra of PURs, the band corresponding to –NCO (2270 cm^−1^) disappeared. Generally, individual spectra of the all polyurethanes were very similar (Figure S1, Supplementary Materials).

**Figure 2:**
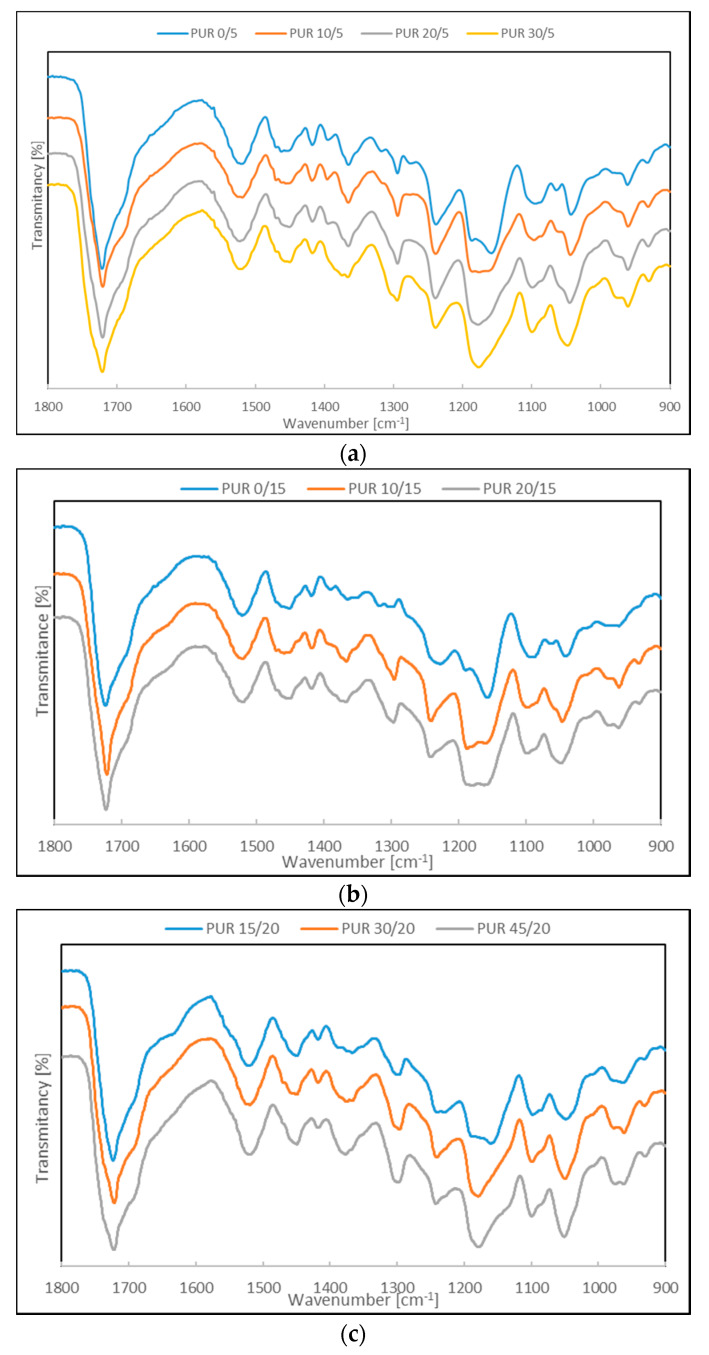
ATR-FTIR spectra in the range 900 cm^−1^ to 1800 cm^−1^ of (**a**) PUR x/5, (**b**) PUR x/15 and (**c**) PUR x/20.

The main differences were observed in the multiple band in the range 1040 cm^−1^ to 1245 cm^−1^ assigned to stretching C–O bonds in soft segments [12] (Figure 2). Shifting these peaks in this range, after adding or increasing the amount of R,S-PHB, indicated that this polyol was successfully incorporated into the PURs structure (Figure S2, Supplementary Materials).

Introducing an amorphous R,S-PHB with a lower distance between carbonyl groups in the chain than PCL into soft segments increased possible interaction between urethane and carbonyl groups. Such an increase in hydrogen bond formation after introducing a substrate with a larger number of hydrogen bonding sites was already observed [13]. In consequence, more hydrogen bonded –N–H groups were formed and the band of their stretching vibration (around 3350 cm^−1^) shifted to a lower wavenumber (Table S1, Supplementary Materials).

The intensity of the C–H stretching vibration around 2920 cm^−1^ (Figure S1, Supplementary Materials) was used as the standard against which the intensity of the urethane carbonyl bands at 1720 cm^−1^ was compared. As the length of the repeating mer in R,S-PHB was shorter than in PCL, the frequency of arising urethane groups and the amount of carbonyl ester groups were greater in polyurethane structure after R,S-PHB using for the synthesis of soft segments. This caused the intensity of the carbonyl of PUR x/5 to increase with increasing amounts of R,S-PHB. This indicated the presence of R,S-PHB on the polyurethane surface. However, after using a higher amount of PCL_t_ for PUR x/15 and PUR x/20 building, the changes in peak intensity were not so straightforward. The reason was probably the inhibition of chain mobility after increasing the number of crosslinks.

**Figure 3:**
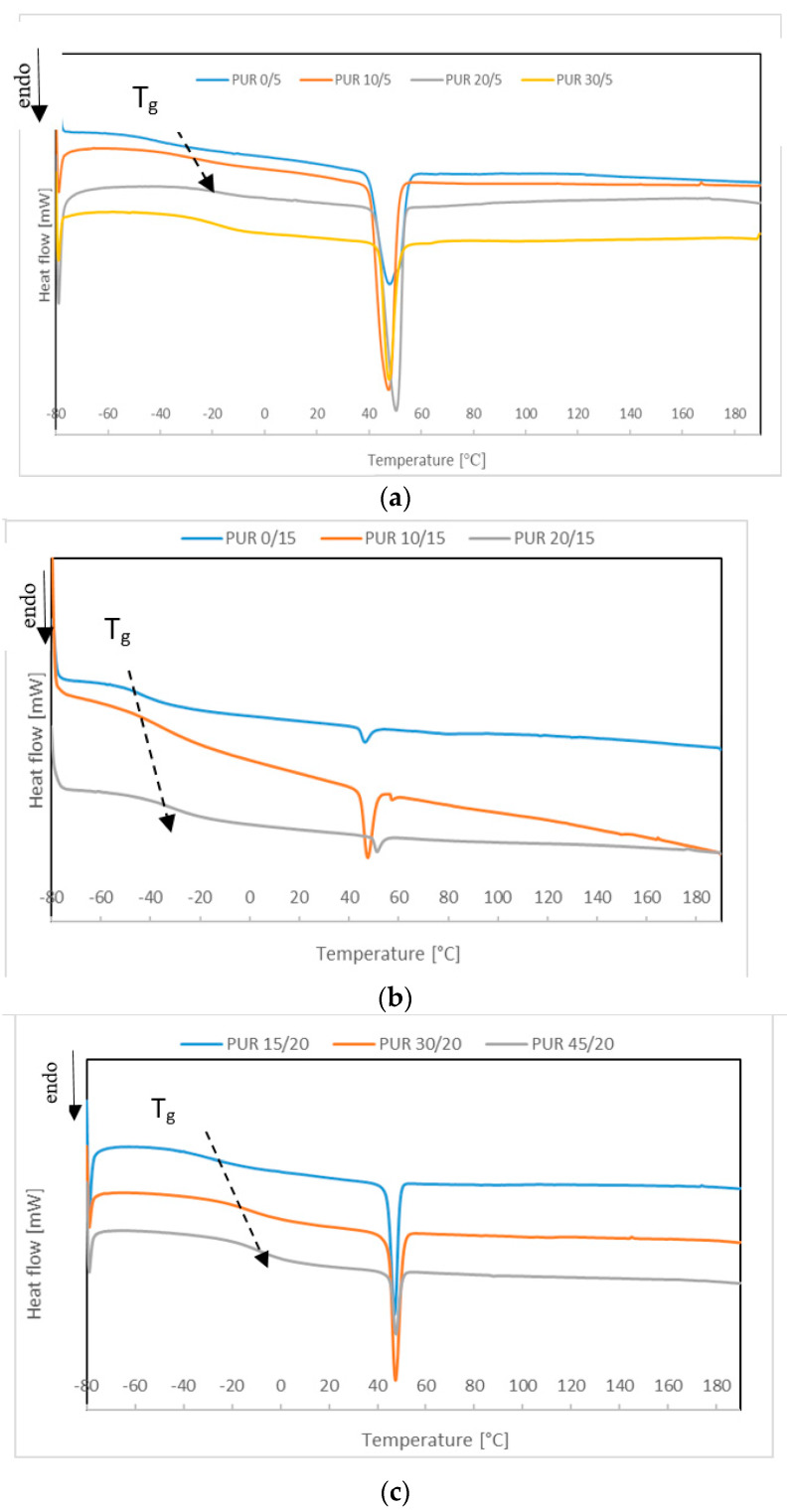
DSC thermograms of the first heating scan of (**a**) PUR x/5; (**b**) PUR x/15; and (**c**) PUR x/20.

Crystallinity of the synthetized PURs was low. Only PURs based on 5 wt % of PCL_t_ in soft segments had the pronounced melting peak with significant melting enthalpy (Figure 3). Increasing R,S-PHB in the soft segments structure of PURs with the higher amount of PCL_t_ increased discreetly melting enthalpy. Despite the amorphous nature of R,S-PHB, its presence in the polymer network facilitated PCL chains moving and forming into crystalline forms. After the ordering of PCL chains, they were stabilized by hydrogen bonds formed due to R,S-PHB presence. Next, these ordered chains were formed into crystalline forms, which was the reason the melting enthalpy increased for PURs with intermate quantities of R,S-PHB present. However, if the amount of R,S-PHB was even greater, its amorphous nature caused the soft segment to become more amorphous. It could be seen that the *T*_m_ also slightly increased (Table 3).

Glass transition temperature of soft segments was significantly higher after increasing of R,S-PHB in the PURs structure (Figure 3). The biggest changes were observed for PURs with the highest number of branching points. As glass temperature of oligomer R,S-PHB was −5.5 °C, its blending with PCL chains increased *T*_g_ of soft segments. Presence of the one *T*_g_ on DSC thermograms indicated on miscibility of these two oligomerols and increasing the R,S-PHB quantity increased the glass transition temperature.

Since the DSC thermograms from the first heating cycle show all changes occurring in polymer structure during sample formation and subsequent storage, it was assumed that lower *T*_g_ PUR x/15 compared to PUR x/5 might result from differences in conditions (e.g., conditioning temperature). However, the results from the second heating cycle clearly showed that the addition of more PCL_t_ caused cross-linking of the chains and thus their stiffening, which increased the *T*_g_^II^ temperature.

The DSC investigation was conducted about 2 months after the synthesis of PURs. During this time, polyurethane chains slowly reorganized and ordered, resulting in the formation of crystalline forms. Therefore, after cooling a PURs sample at the rate of 10 °C·min^−1^, it was found out that no crystalline forms were formed in the polymer network. On thermograms of the second heating cycle, only glass transition of the soft segments was observed. Generally, the values of *T*_g_ from the second heating scan were lower than the ones obtained from the first heating. Only in the case of PUR 0/15 and PUR 20/15, whose melting enthalpy of soft segments was less than 1 J/g, *T*_g_ from the second heating was higher. It was concluded that the presence of crystalline forms hindered mobility of chains, which consequently moved *T*_g_ to higher temperatures (Table 3).

The analysis of the results of the second heating (after eliminating the thermal history of a sample) confirmed compliance with the results presented in [11], which indicated that an increase in *M*_c_ resulted in an increment of *T*_g_. PURs with the small amount of PCL_t_ and M_c_ about 24,000 g·mol^−1^ had lower *T*_g_^II^ than PURs with higher branching points. Nevertheless, it was concluded that side chains CH_3_ and C_2_H_5_ were too short to have a plasticizing effect, as it was stated in [11] in the case of side chains in triolein used for PUR synthesise.

**Figure 4:**
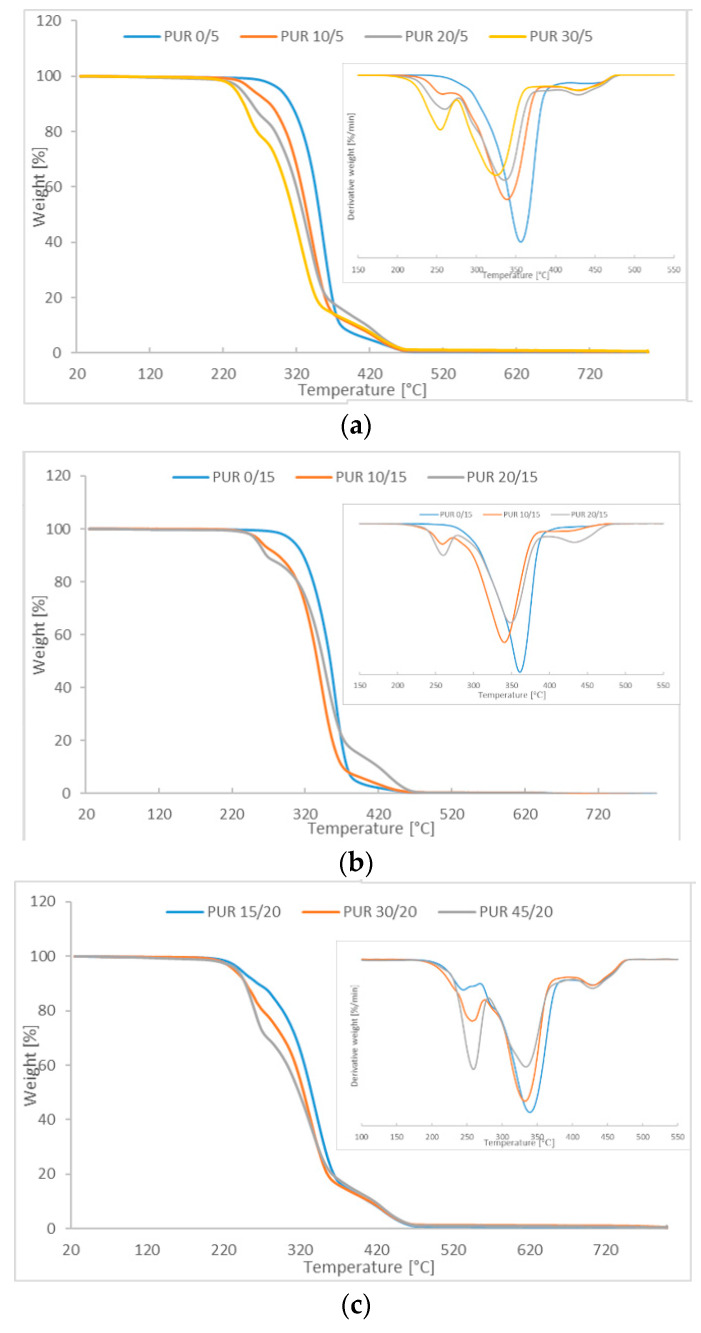
TGA and DTG curves of (**a**) PUR x/5, (**b**) PUR x/15; and (**c**) PUR x/20.

Thermal decomposition of PUR was measured by TG and DTG (Figure 4). In the case of PURs without R,S-PHB a one-step process of thermal degradation was observed. Temperature of the maximum degradation of PUR 0/5 and PUR 0/15 was 356 °C, and 362 °C, respectively (Table S3, Supplementary Materials). The initial degradation temperature was higher for PUR 0/15 with the increased number of cross-links, which is in agreement with the literature data [14,15]. Increasing the amount of PCL_t_ in the structure of soft polyurethane segments, without R,S-PHB clearly increasing the thermal stability of the sample, indicated an increase in cross-linking. However, the addition of R,S-PHB disrupted cross-linking processes. The lower *T*_i_ and *T*_5_ values of polyurethanes with a higher amount of PCL_t_ compared to PUR x/5 indicated that the polyurethane network had chains with low molecular weights that underwent thermal degradation faster. In addition, the crosslinked networks of these polyurethanes may contain the residual particles of solvent, which further affect the weight reduction during the TG analysis. Derivative TG curves clearly indicated that PURs with R,S-PHB degraded in three stages. The presence of secondary OH groups in the R,S-PHB structure caused this polyol to be less reactive than PCL_d_ and PCL_t_ in reaction with the isocyanate group [16]. The reactivity of the secondary group is assumed to be only 30% of that of primary alcohols [17]. In consequence distribution of molecular weight was therefore probably high. The resulting shorter chains probably degraded at a lower temperature than chain with the high molecular weight and the initial temperature (*T*_i_) of thermal degradation of PURs with R,S-PHB was lower (Table 3). Such a decrease of degradation temperature with the molecular weight decreasing was observed for poly(dimethylsiloxane) [18] and poly(methyl methacrylate) [19]. Knowing that degradation of PUR starts from a breakage of urethane bonds in hard segments [20], there was a higher intensity of the first peak of the synthetized polyurethanes with R,S-PHB, as the presence of R,S-PHB caused formation of more urethane groups, and temperatures of the initial decomposition and reduction of 5% and 10% samples mass were found to be the lowest. Polyurethanes based on high amount of R,S-PHB (PUR 30/5, PUR 30/20 and PUR 45/20) underwent the fastest thermal degradation (*T*_i_ was the lowest), despite the high number of cross-linking nodes (in case of PUR 30/20 and PUR 45/20). The number of side (CH_3_ and C_2_H_5_) chains in PUR 45/20 structure was the highest (Table 1). Their presence caused the macrochains to move away from one another, and as a consequence, the interaction between them was reduced, so the thermal stability was also low. 

**Figure 5:**
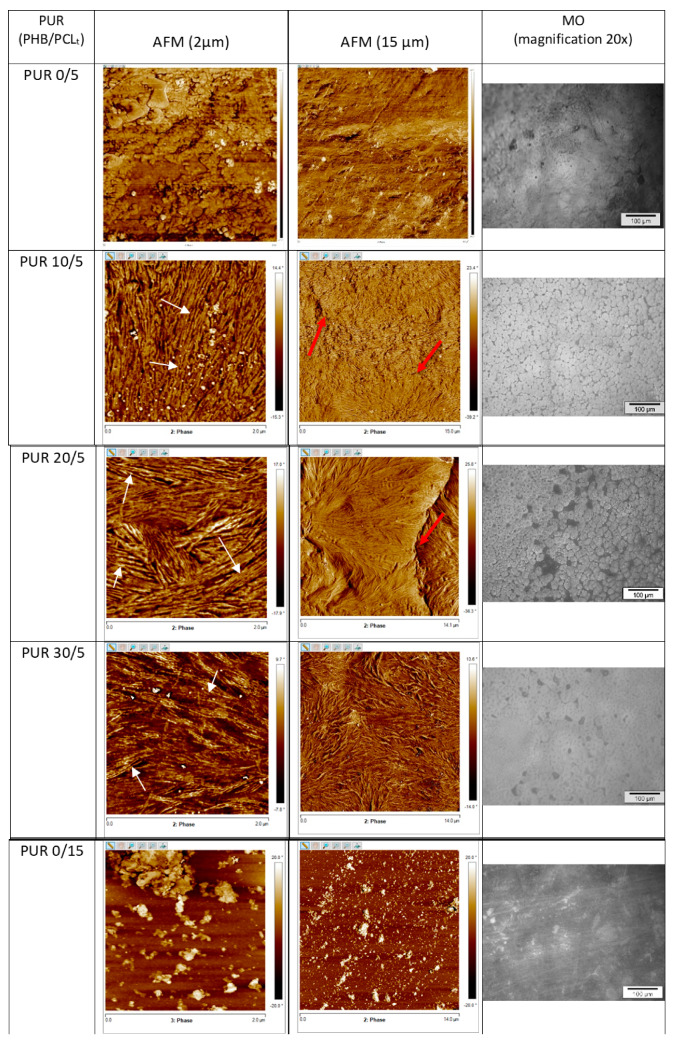

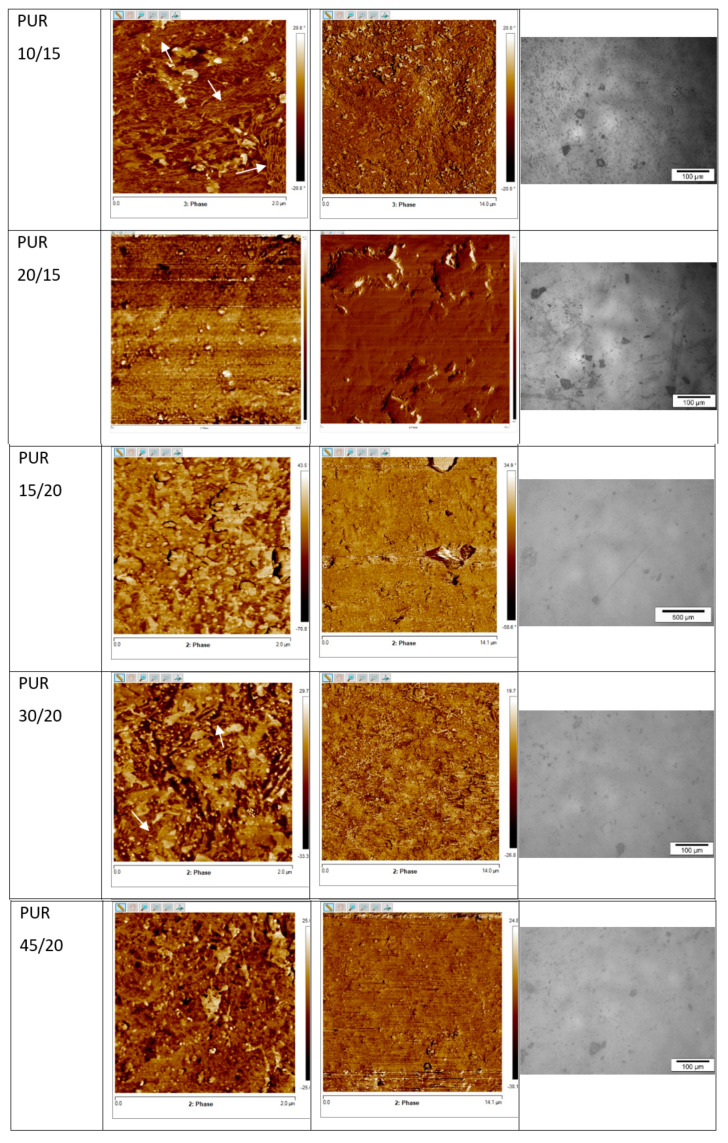
AFM phase and MO images of surface of branched polyurethanes (images for PUR 10/5 and PUR 20/5 were presented in the paper [9]).

On the surface of the samples with the highest enthalpy of melting of soft segments (Table 3), the lamellas (marked with white arrows in Figure 5) that could organize into spherules were visible. The spherules and a clear boundary between them on AFM images of PUR 10/5 and PUR 20/5 were marked with red arrows in Figure 5. The width of these lamellas was within 10–40 nm. Cross-sectional images indicated that lamellas occurred also throughout the entire sample volume (Figure S2, Supplementary Materials). 

The PUR 0/5 surface was irregular, with inclusions of 700 nm × 500 nm (large inclusions), 140 nm × 220 nm (medium inclusions) and 60 nm × 70 nm–90 nm × 130 nm (fine inclusions) (Figure 5). On the surface of PURs with the larger amount of PCL_t_ in soft segments, a few inclusions and the small number of lamellase (marked with white arrows) of the width of 10–20 nm were observed (e.g., PUR 0/15 and PUR 30/20). However, they were not arranged into any crystalline forms, as it was confirmed by the DSC investigation (low melting enthalpy was observed for these polymers) (Table 3).

As it is well-known, darker zones in the AFM phase images correspond to the amorphous phase of PURs, while brighter zones correspond to the crystalline phase of these PURs. Taking this into consideration, the AFM results can be correlated with the DSC results [21], the AFM phase images of polyurethanes indicate that PUR x/15 samples were the least crystalline material.

Introducing R,S-PHB into the structure of PURs with low branching points (PUR x/5) significantly reduced root mean square roughness (R_q_) from 85 nm for PUR 0/5 to even 14 nm in the case of PUR 30/5 (Table S4, Supplementary Materials). The effect of R,S-PHB on roughness was much smaller in the case of PURs with more branching nodes.

Images of the surface in the microscopic scale (under the optical microscope) were in good agreement with AFM observations. On the surface of PUR x/5 with tendency to crystallisation, the characteristic circular objects were visible. Surface topography of other polyurethanes was smooth with no pores or protrusions, but with fine, heterogeneous inclusions. 

**Figure 6:**
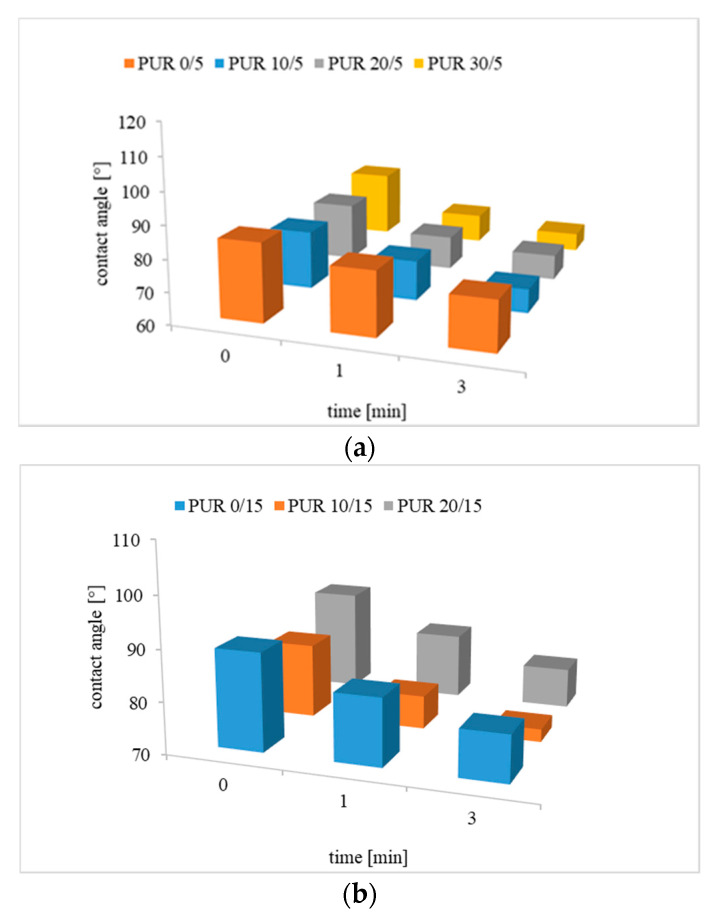

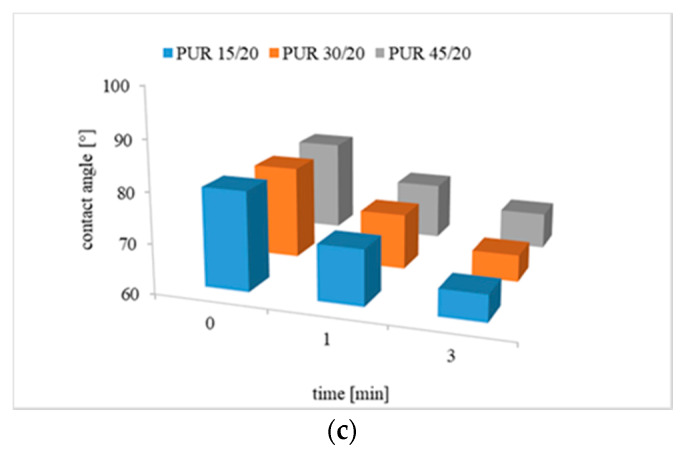
Contact angle of (**a**) PUR x/5; (**b**) PUR x/15; and (**c**) PUR x/20 0, 1 and 3 min after immersion of a water drop on the sample surface.

The contact angle of the synthetized polyurethanes was high, near hydrophobic materials. However, as depicted in Figure 6 the contact angle observed after 3 min from immersing of a water drop on the polymer surface was significantly reduced. This phenomenon was observed also for polyurethanes without R,S-PHB, but changes in these case were lower. This indicated affinity of these polymers to water. 

Samples with higher roughness should supposedly have a lower contact angle [22]. However, the obtained results did not confirm this. Also, the expected increase in water contact, due to the increased cross-links, was not found [23]. An increase in the amount of PCL_t_ in the structure of soft segments (at the same time reduction in M_c_) from 5 wt % to 15 wt % increased the contact angle, whereas a further increase in the amount of triol reduced it (Figure 6 and Table S4). The highest contact angle was observed for the second series of PUR with 15 wt % PCL_t_. It was clearly seen that polyurethanes without R,S-PHB were characterized by the highest contact angle. 

It is believed that after film formation, on the sample contact surface with air, were mainly PCL chains because of their hydrophobicity. However, as it was said before, after 3 min from the placement of the water drop, the angle decreased, which indicated an increase in the affinity of polyurethanes to water. These changes were higher for PURs with R,S-PHB. This was due to the higher hydrophilicity of the R,S-PHB chains than PCL, as there was more carbonyl groups capable to forming hydrogen bonds with water in R,S-PHB. Thus, hydrophilic R,S-PHB chains migrated to the sample surface under the influence of water droplets.

In case of almost linear PUR x/5, the ordering of chains and crystallinity was the highest, and the free migration of PCL chains to the surface was limited. The presence of R,S-PHB on PURs surface was observed by ATR-FTIR analysis. In contrast, the interaction in PUR x/15 between the chains was much smaller (which was confirmed by the DSC results) due to greater number of branches so the hydrophobic PCL chains could easily move to the surface of the sample, and the contact angle increased. 

However, with the highest quantity of PCL_t_, polyurethanes were cross-linked, which resulted in reduced polycaprolactone chains mobility during the sample formation, hence the reduction of the contact angle to values similar to PUR x/5.

The water affinity of the obtained polyurethanes, observed during the contact angle measurements, was confirmed by a high water uptake. Water absorption percentage visibly increased with an increase in immersion time for all samples, and despite of 21 days of incubation, some samples did not obtain the equilibrium swelling.

**Figure 7:**
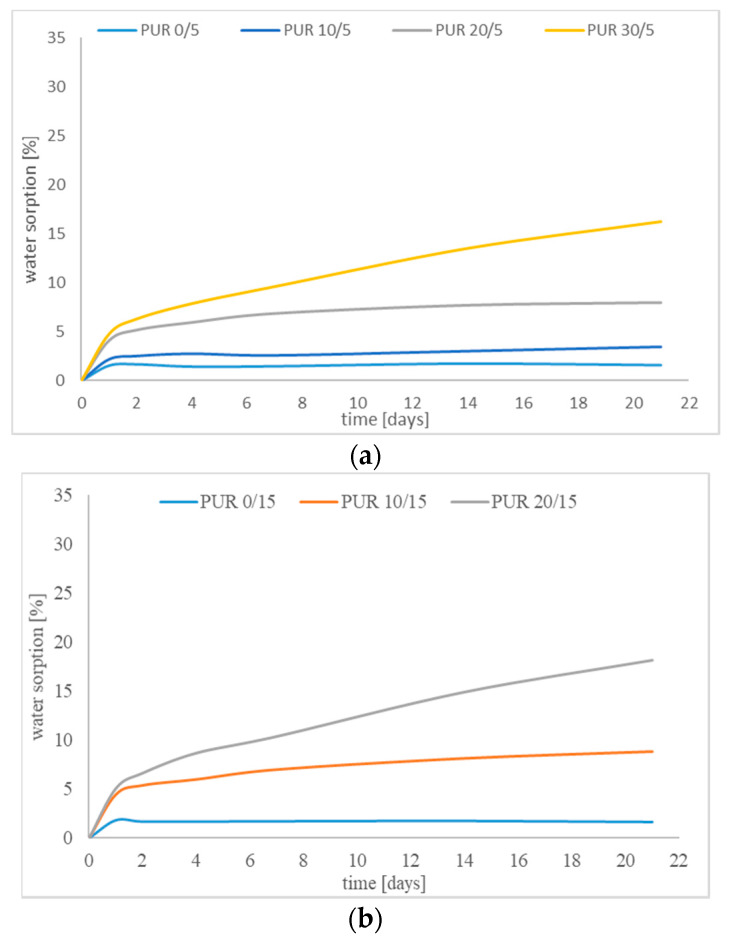

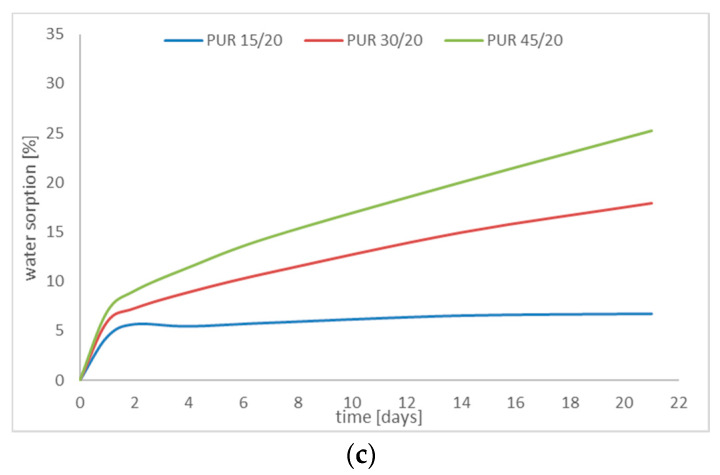
Water sorption of (**a**) PUR x/5; (**b**) PUR x/15; and (**c**) PUR x/20.

The samples of polyurethanes without R,S-PHB increased their weight only by 1.6% after the first day of incubation in water, and this value did not change during investigations (Figure 7). Undoubtedly, addition of R,S-PHB into the structure of soft segments caused a significant increase in water sorption. The same tendency was observed by Wang and others [24]. Moreover, the greater the amount of R,S-PHB in the polyurethane network, the greater water sorption. The highest amount of water was absorbed for PUR 45/20 polymer with the soft segments built in almost half with R,S-PHB. 

Samples with 5 wt % of PCL_t_ in soft segments absorbed the lowest amount of water. The highest *M*_c_ of chains between branch points was connected with the highest possibility of interaction (such as hydrogen bonds) between long aliphatic polyurethane chains and water molecules hardly penetrated inside the bulk sample. As the content of side CH_3_ groups increased (Table 1), those interactions were inhibited, which facilitated penetration of water into the polyurethane network. Meanwhile, when the amount of PCL_t_ increased, the polymer with large free spaces, limited through cross-linking, was obtained. Relatively long chains between the network nodes (high *M*_c_) caused that water could penetrate. Hence, an increase in water sorption was observed in the case of PUR x/15. However, when the number of cross-links was too high (as in the case of PUR x/20), water molecules could not penetrate deep into the network.

**Figure 8:**
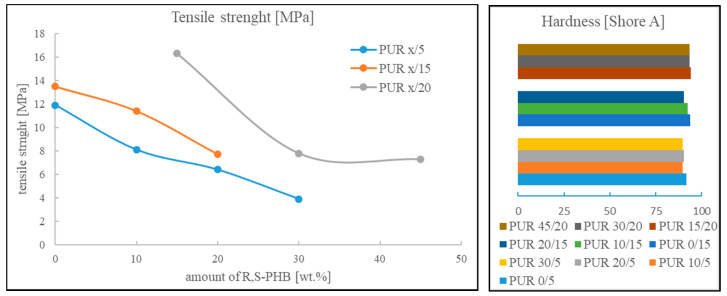
Dependence of tensile strength on the amount of R,S-PHB in the structure of polyurethanes and their hardness.

The relation between tensile strength and the amount of R,S-PHB in the structure of soft segments and values in samples hardness are depicted in Figure 8.

Tensile strength values were not high, but they were still higher than for example in the case of polyurethane composite with natural PHB used as reinforcement [25]. Two significant parameters affected tensile strength: The number of cross-links (introduced with PCL_t_) and the amount of R,S-PHB in soft segments. In all PUR series there was a trend of a decrease in tensile strength with an increase of R,S-PHB in the network (Figure 8). It was supposed that the reason was the presence of a secondary group in the R,S-PHB chain, which hindered the reaction with NCO groups; and consequently, the structure was not uniform and statistically repeatable. This caused worse ordering of individual segments, and thus, reduced the interaction between chains and deteriorated mechanical properties. Wang and others found that by introducing a higher amount of natural polyhydroxybutarate diol (also with a secondary hydroxyl group), the tensile strength of polyurethanes proved to be higher [24]. Thus, the amorphous R,S-PHB affect the PURs properties in different way than crystalline R-PHB. We suppose that the crystalline nature of natural PHB could be the reason this increased strength.

The number of branching nodes was another considered factor that affected mechanical strength. Increasing the amount of cross-linking by using more quantity of PCL_t_ and reducing M_c_, between cross-linking nodes, increased tensile strength of polyurethanes, which was expected [26].

The hardness of PURs samples was high and slightly decreased after R,S-PHB adding. At the same time hardness of samples increased after increasing the amount of triol (Figure 8 and Table S5, Supplementary Materials). The increasing of polyurethane hardness with increasing in cross-links number is well known [26]. However, it was expected that differences between hardness of branched PURs and samples with higher amount of PCL_t_ would be more significant. Moreover, hardness of the samples with low branching nodes should be lower. On the other hand, the highest PUR x/5 crystallinity was antagonistic to the effect of the highest linearity of these polyurethanes and, consequently, hardness did not degrease as much, as it was expected. 

## 4. Conclusion

In this work the results of physic-chemical, structural, thermal and sorptive properties of branched and cross-linked polyurethanes, based on synthetic poly([R,S]-3-hydroxybutyrate), were described. It was proved that the use of such an amorphous, synthetic analogue of natural PHB and manipulation of the amount of triol (PCL_t_) could provide an opportunity to design the properties of the finished polyurethane. On the whole, introducing R,S-PHB and its further increasing in soft segments of polyurethanes significantly affected their properties: more hydrogen bonds were formed, glass transition temperature of soft segments increased, initial thermal degradation declined, crystallinity was changed, the contact angle decreased, water sorption raised and tensile strength decreased.

Meanwhile, increasing in the amount of PCL_t_ in soft segments increased the number of short side chains and branch/cross-links nodes, with reduced M_c_ between them. These factors affected the glass transition temperature and melting enthalpy, which increased after adding more than 5 wt % of triol into soft segments. It also facilitated the water sorption, but only to a certain PCL_t_ value (PUR x/15), as further increases in its quantity (in the case of PUR x/20) caused a significant increase in the number of cross-linking nodes, which made it difficult for water molecules to penetrate. The mechanical properties increased after increased PCL_t_.

To sum up, it was found out that by modifying the amount of polycaprolactone triol and poly([R,S]-3-hydroxybutyrate) in soft segment, a polyurethane material with the assumed thermal, sorption and mechanical properties could be obtained. However, linear changes in the properties of polyurethanes with increase PCL_t_ could only be predicted to a certain limited value of the amount of triol, because then they were affected by too many cross-links.

## Figures and Tables

**Table 1:** Chemical composition of PURs.

PUR (PHB/PCL_t_)	Percentage of Oligomerol in Soft Segments (wt %)			M_c_ (g/mol)	Side CH_3_ Content (%)	Side C_2_H_5_ Content (%)
	R,S-PHB	PCL_t_	PCL_d_			
PUR 0/5	0	5	95	24,228.1	0	0.6
PUR 10/5	10	5	85	24,464.5	1.1	0.6
PUR 20/5	20	5	75	24,401.6	2.2	0.6
PUR 30/5	30	5	65	24,095.2	3.3	0.6
PUR 0/15	0	15	85	8508.9	0	1.6
PUR 10/15	10	15	75	8503.8	1.1	1.6
PUR 20/15	20	15	65	8068.8	2.2	1.6
PUR 15/20	15	20	65	6048.6	1.6	2.2
PUR 30/20	30	20	50	6041.9	3.3	2.2
PUR 45/20	45	20	35	6032.6	5.0	2.2

**Table 2:** Characteristic of polyurethanes.

PUR (PHB/PCL_t_)	Density [g/cm^3^]	Oil Sorption [%]	Transparency	Solubility/swelling in DMF
PUR 0/5	1.13	0.5	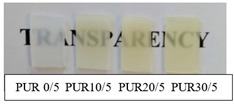	soluble
PUR 10/5	1.14	0.6		
PUR 20/5	1.10	0.8		
PUR 30/5	1.13	0.8		
PUR 0/15	1.10	0.7	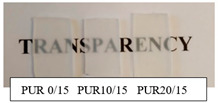	swelled
PUR 10/15	1.13	0.8		
PUR 20/15	1.10	0.6		
PUR 15/20	1.13	0.6	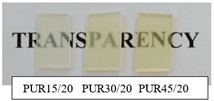	swelled
PUR 30/20	1.12	0.6		
PUR 45/20	1.13	0.8		

**Table 3:** Results of the DSC and TGA analysis of polyurethanes.

PUR (PHB/PCL_t_)	*T*_g_^I^ (°C)	*T*_g_^II^ (°C)	*T*_m_ (°C)	ΔH [J/g]	*T*_i_ (°C)	*T*_5%_ (°C)	*T*_10%_ (°C)	*T*_f_ (°C)
PUR 0/5	−43.1	−46.6	47.8	20.9	267.4	287.3	295.3	467.6
PUR 10/5	−24.5	−42.2	47.5	27.4	243.5	265.9	285.8	453.3
PUR 20/5	−18.0	−36.4	50.4	25.8	231.3	249.5	264.2	458.1
PUR 30/5	−19.7	−28.9	47.7	12.8	223.3	240.7	250.9	457.4
PUR 0/15	−45.3	−41.8	46.2	0.9	278.0	303.2	316.1	450.0
PUR 10/15	−36.6	−38.8	47.3	4.9	240.1	258.0	278.1	450.3
PUR 20/15	−33.0	−28.3	51.1	0.9	238.3	253.1	266.4	460.0
PUR 15/20	−39.4	−42.5	47.1	7.7	227.2	244.8	268.4	456.7
PUR 30/20	−11.5	−17.7	47.5	10.1	221.9	237.9	251.7	458.1
PUR 45/20	−8.9	−7.2	47.7	3.6	223.2	242.0	248.7	457.5

*T*_g_^I^—glass transition temperature of soft segments from DSC thermograms of 1^st^ heating scan; *T*_g_^II^—glass transition temperature of soft segments from DSC thermograms of 2^nd^ heating scan; T_m_—melting temperature of soft segments from DSC thermograms of 1^st^ heating scan; ΔH—melting enthalpy of soft segments from DSC thermograms of 1^st^ heating scan; *T*_i_—initial decomposition temperature of samples (1% degradation of samples); *T*_5%_—temperature of 5% degradation of samples; *T*_10%_—temperature of 10% degradation of samples; *T*_f_—final decomposition temperature of samples (corresponds to 1% residual dry mass of sample after thermal degradation).

## Supplementary Materials

The following are available online at https://www.mdpi.com/2073-4360/12/5/1068/s1, Figure S1: ATR-FTIR spectra of PURs, Figure S2: AFM cross-section images of PURs, Table S1: ATR-FTIR bands of polyurethanes in the range of 4000 to 1200 cm^−1^, Table S2: ATR-FTIR bands of polyurethanes in the range of 1300 to 1000 cm^−1^, Table S3: Degradation temperature (*T*_degr_) at three stages of degradation from DTG curves, Table S4: Root mean square roughness (R_q_) and average roughness (R_a_) of AFM and contact angle of PURs, Table S5: Hardness of the obtained polyurethanes.
