# Supplementary material for: Predicted Studies of Branched and Cross-Linked Polyurethanes Based on Polyhydroxybutyrate with Polycaprolactone Triol in Soft Segments
Source: Polymers (Basel). 2020 May 7;12(5):1068. doi: 10.3390/polym12051068 (PMC7284939; doi:10.3390/polym12051068)
Supplement: Supplementary file 1 [file polymers-12-01068-s001.pdf]

polymers

MDPI

# Predicted studies of branched and cross-linked polyurethanes based on polyhydroxybutyrate with polycaprolactone triol in soft segments

**Joanna Brzeska[1*], Agnieszka Tercjak[2], Wanda Sikorska[3*], Marek Kowalczuk[3], Maria Rutkowska[1]**

[1] *Department of Commodity Industrial Science and Chemistry, Gdynia Maritime University, 83 Morska Street, 81-225 Gdynia, Poland*
[2] *University of the Basque Country (UPV/EHU), Department of Chemical and Environmental Engineering, Group 'Materials+Technologies' (GMT), Plaza Europa 1, 20018 Donostia-San Sebastián, Spain*
[3] *Centre of Polymer and Carbon Materials, Polish Academy of Sciences, 34 M. Curie-Sklodowska Street, 41-819 Zabrze, Poland*

**j.brzeska@wpit.umg.edu.pl*, **wsikorska@cmpw-pan.edu.pl*

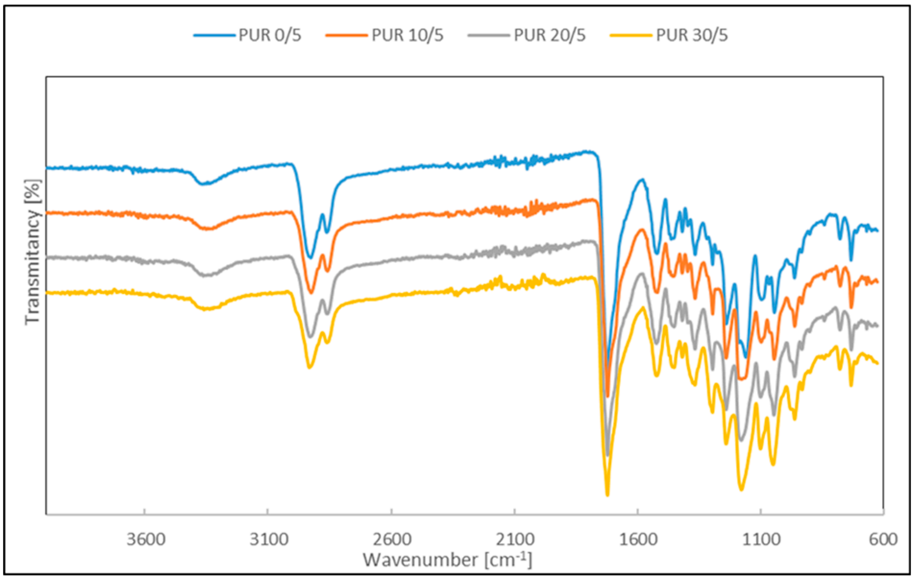


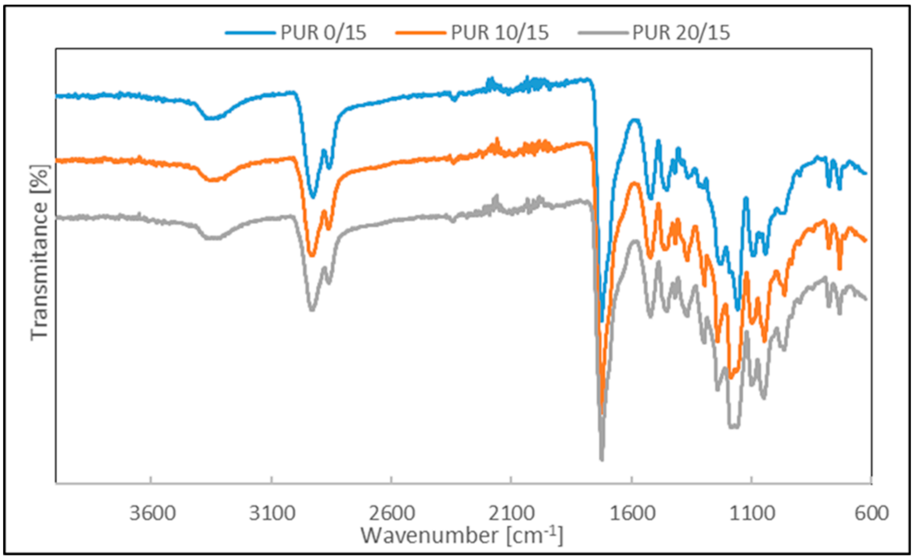

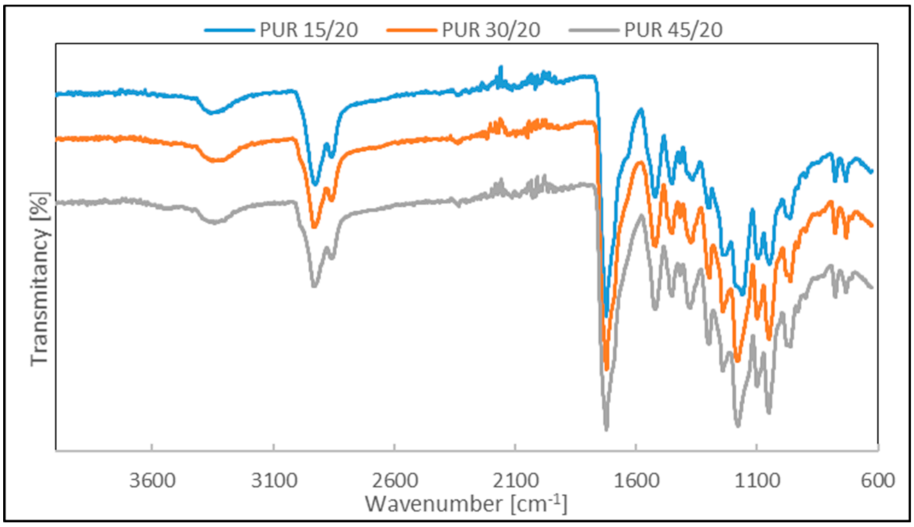


**Figure S1. ATR-FTIR spectra of PURs**

**Table S1. ATR-FTIR bands of polyurethanes in the range of 4000 cm$^{-1}$ to 1200 cm$^{-1}$.**

| **Sample** | **–N-H stretching vibration [cm$^{-1}$]** | **–C=O stretching vibration [cm$^{-1}$]** | **N–H bending vibr. and C–N stretching vibr. (amide II band) [cm$^{-1}$]** | **C–N stretching vibr. (amide III band) [cm$^{-1}$]** |
|---|---|---|---|---|
| PUR 0/5 | 3366.9 | 1721.7 | 1520.8 | 1238.7 |
| PUR 10/5 | 3351.4 | 1720.7 | 1518.6 | 1239.2 |
| PUR 20/5 | 3363.0 | 1720.9 | 1522.1 | 1239.5 |
| PUR 30/5 | 3360.6 | 1721.2 | 1521.8 | 1239.3 |
| PUR 0/15 | 3359.2 | 1723.7 | 1520.7 | 1227.9 (overlept) |
| PUR 10/15 | 3354.3 | 1721.5 | 1519.8 | 1240.9 |
| PUR 20/15 | 3360.4 | 1722.8 | 1520.0 | 1241.0 |
| PUR 15/20 | 3359.7 | 1720.4 | 1520.3 | 1239.6 |
| PUR 30/20 | 3316.3 | 1721.6 | 1519.2 | 1241.4 |
| PUR 45/20 | 3342.6 | 1722.4 | 1519.0 | 1242.5 |

**Table S2. ATR-FTIR bands of polyurethanes in the range of 1300 cm$^{-1}$ to 1000 cm$^{-1}$.**

| Sample | Multiplet band of C–O bonds in soft segments [cm$^{-1}$] | | | |
|---|---|---|---|---|
| PUR 0/5 | 1238.7 | 1158.5 (+ small 1185,0) | 1095.8 | 1043.4 (+ small 1063.50) |
| PUR 10/5 | 1239.2 | 1176.2 | 1096.8 | 1044.3 (+ small 1058.7) |
| PUR 20/5 | 1239.5 | 1177.7 | 1099.3 | 1045.3 |
| PUR 30/5 | 1239.3 | 1177.3 | 1099.8 | 1048.0 |
| PUR 0/15 | 1227.3 | 1156.9 (+ small 1184.6) | 1088.5 | 1041.3 (+ small 1060.7) |
| PUR 10/15 | 1240.9 | 1187.0 (+ small 1161.4) | 1097.5 | 1045.9 (+ small 1056.3) |
| PUR 20/15 | 1241.0 | 1181.6 (+ small 1157.1) | 1098.1 | 1048.0 |
| PUR 15/20 | 1239.6 | 1161.0 (+ small 1196.0) | 1097.8 | 1048.6 |
| PUR 30/20 | 1241.4 | 1179.3 | 1099.0 | 1050.0 |
| PUR 45/20 | 1242.5 | 1177.9 | 1099.4 | 1050.9 |

**Table S3. Degradation temperature ($T_{degr}$) at three stages of degradation from DTG curves**

| Sample | $T_{degr1}$ [°C] | $T_{degr2}$ [°C] | $T_{degr3}$ [°C] |
|---|---|---|---|
| PUR 0/5 | - | 356.2 | - |
| PUR 10/5 | 256.8 | 339.6 | 430.9 |
| PUR 20/5 | 258.1 | 337.0 | 429.2 |
| PUR 30/5 | 255.5 | 325.6 | 429.0 |
| PUR 0/15 | - | 361.8 | - |
| PUR 10/15 | 260.0 | 350.8 | 422.2 |
| PUR 20/15 | 260.0 | 350.8 | 433.4 |
| PUR 15/20 | 247.0 | 339.5 | 429.4 |
| PUR 30/20 | 257.8 | 335.2 | 429.2 |
| PUR 45/20 | 260.0 | 333.9 | 429.0 |

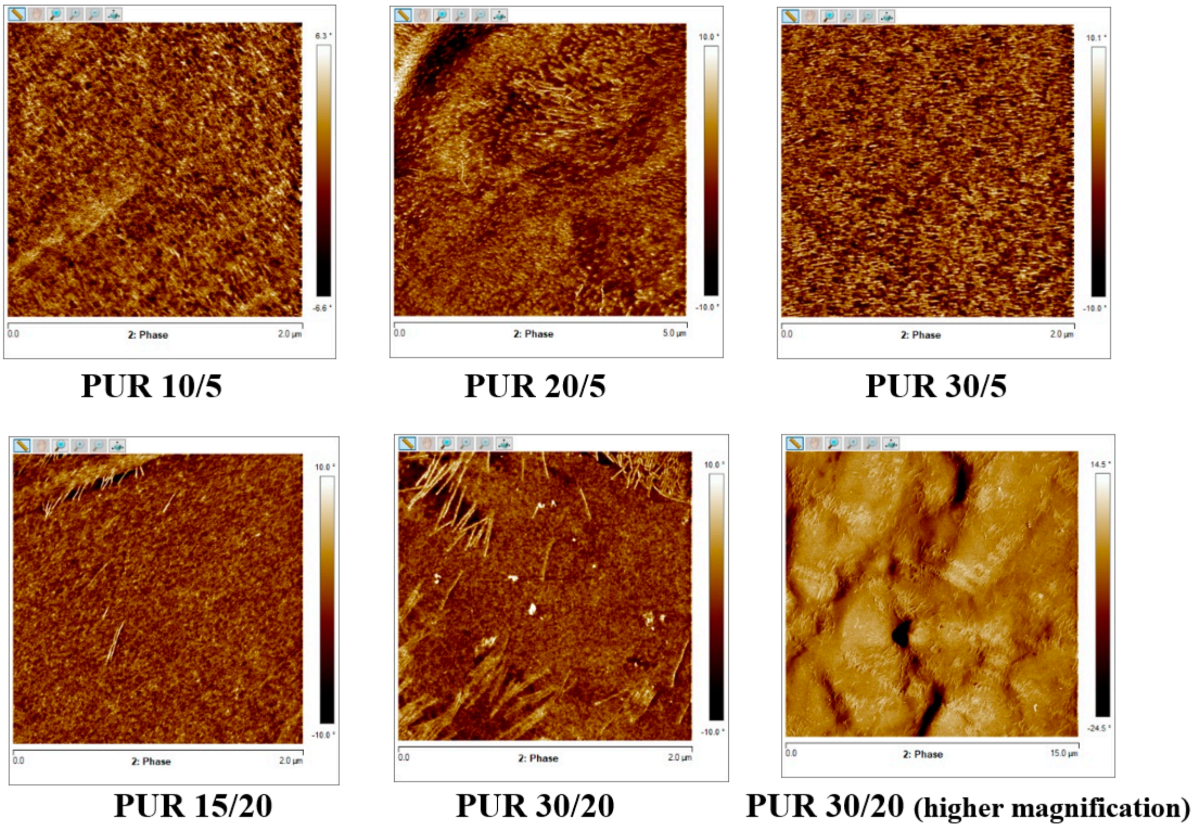


**Figure S2. AFM cross-section images of PURs**

**Table S4. Root mean square roughness (Rq) and average roughness (Ra) of AFM and contact angle of PURs**

| Sample | Contact angle [°] | | | Root mean square roughness ($R_q$) [nm] | Average roughness ($R_a$) [nm] |
|---|---|---|---|---|---|
| | 0 min | 1 min | 3 min | | |
| PUR 0/5 | 84.5±0.9 | 79.9±1.5 | 75.5±0.8 | 85 | 67 |
| PUR 10/5 | 78.2±6.3 | 72.4±7.3 | 67.5±3.0 | 28 | 19 |
| PUR 20/5 | 78.2±4.1 | 70.5±3.8 | 67.9±0.2 | 20 | 16 |
| PUR 30/5 | 80.9±1.6 | 69.1±2.1 | 65.7±2.6 | 14 | 11 |
| PUR 0/15 | 89.1±3.3 | 83.1±3.3 | 79.1±3.6 | 10 | 8 |
| PUR 10/15 | 84.5±0.9 | 76.5±3.0 | 72.5±3.0 | 16 | 7 |
| PUR 20/15 | 89.8±1.8 | 82.8±2.6 | 77.8±2.5 | 20 | 17 |
| PUR 15/20 | 79.9±2.0 | 71.1±2.3 | 65.3±3.1 | 21 | 16 |
| PUR 30/20 | 78.6±1.6 | 71.1±2.3 | 65.3±3.1 | 20 | 14 |
| PUR 45/20 | 78.6±2.5 | 71.4±4.6 | 67.3±4.9 | 18 | 14 |

**Table S5. Hardness of the obtained polyurethanes**

| **Sample** | **Hardness [°Shore A] ± SD** |
|---|---|
| PUR 0/5 | 91.7 ± 1.3 |
| PUR 10/5 | 89.5 ± 0.1 |
| PUR 20/5 | 90.4 ± 1.0 |
| PUR 30/5 | 89.5 ± 2.6 |
| PUR 0/15 | 93.7 ± 0.7 |
| PUR 10/15 | 92.4 ± 1.0 |
| PUR 20/15 | 90.4 ± 5.8 |
| PUR 15/20 | 94.1 ± 3.0 |
| PUR 30/20 | 93.3 ± 1.8 |
| PUR 45/20 | 93.4 ± 0.6 |
